# Cervical Epidural Steroid Injection: Parasagittal versus Midline Approach in Patients with Unilateral Cervical Radicular Pain; A Randomized Clinical Trial

**DOI:** 10.29252/beat-070208

**Published:** 2019-04

**Authors:** Masoud Hashemi, Payman Dadkhah, Mehrdad Taheri, Kasra Dehghan, Rohollah Valizadeh

**Affiliations:** 1 *Department of Anesthesiology, Fellowship in Pain Management, Anesthesiology Research Center, Shahid Beheshti University of Medical Sciences, Tehran, Iran*; 2 *Department of Anesthesiology, In-Training Fellow of Pain Management, Anesthesiology Research Center, Shahid Beheshti University of Medical Sciences, Tehran, Iran*; 3 *Department of Epidemiology, Student Research Committee, Iran University of Medical Sciences, Tehran, Iran*

**Keywords:** Injections, Epidural, Pain management, Upper extremity, Treatment outcome, Radiculopathy, Intervertebral disc disease

## Abstract

**Objective:**

To compare parasagittal interlaminar cervical epidural steroid injection (PSIL-CESI) and the classic midline interlaminar cervical epidural steroid injection (MIL-CESI) in terms of pain relief and functional improvement in patients with unilateral upper extremity radicular pain.

**Methods:**

This was a randomized clinical trial being conducted in a single pain center in Tehran. Twenty-six patients were allocated into two groups of 13, undergoing either PSIL-CESI or MIL-CESI. After confirmation of radiocontrast spread in the epidural space by fluoroscopic guidance, dexamethasone 8 mg and bupivacaine 0.125% in a volume of 5 ml were delivered to the epidural space. Evaluation of functional state and pain intensity before and 1 month after the procedure was accomplished using the neck disability index (NDI) and the numeric rating scale (NRS) respectively.

**Results:**

Demographic and baseline characteristics of the cases showed no significant statistical difference. Improvements in the NDI and the NRS were observed in both groups; meanwhile, improvements were more pronounced in the PSIL-CESI group as compared to the MIL-CESI group (P<0.001). With the PSIL approach the ventral spread of radiocontrast was significantly higher (38%) than with the MIL approach (0.7%) (P<0.001). All patients in PSIL group showed radiocontrast spread ipsilateral to the painful side and all patients in the MIL group showed a midline distribution of radiocontrast.

**Conclusion:**

PSIL-CESI provides superior pain relief and improvement of functional disability in patients with unilateral upper extremity radicular pain in comparison to the classic MIL-CESI.

**Clinical trial registry:**

IRCT20180524039816N1

## Introduction

Cervical epidural steroid injection (CESI) has been used in treatment of radicular upper extremity pain [1-3]. Several reports of success of these procedures in reduction of upper extremity pain from cervical disc herniation have been presented in the literature [4,5]. Although long-term effectiveness of CESI in such instances is debatable, CESI seems a reasonable choice for management of those patients who are reluctant to undergo surgery and those who are not good candidates for surgical interventions [3]. Transforaminal (TF), MIL, PSIL, and paramedian interlaminar (PMIL) routes are among the routes of epidural access [6]. It is believed that the TF route is more target specific and delivers the injectate to a closer vicinity of the pathologic site [1, 7-9]. 

The TF approach may be associated with devastating complications and it is strongly recommended that this procedure be performed under continuous fluoroscopic imaging and with the help of digital subtraction angiography. It is also recommended that in the cervical region only non-particulate steroid be used [7, 10]. Hazards attributed to the TF approach has led to a search for safer techniques [1, 11]. A number of reports support the superior efficacy of the PSIL approach in the lumbar region in treatment of radicular lower extremity pain [11-13]. To our knowledge there has been no reports in the literature comparing the MIL-CESI and the PSIL-CESI. This study aimed to make a comparison between parasagittal interlaminar cervical epidural steroid injection (PSIL-CESI) and the classic midline interlaminar cervical epidural steroid injection (MIL-CESI) in terms of pain relief and functional improvement in patients with unilateral upper extremity radicular pain.

## Materials and Methods

 *Study population*

  This study was a double-blind, parallel-group, randomized controlled clinical trial. The institutional ethics committee approved the trial (IR.SBMU.RETECH.1397.163). The study protocol also was registered by the Iranian registry for clinical trials (IRCT20180524039816N1; www.irct.ir). All patients were thoroughly informed regarding the study procedure and written informed consent was acquired from all participating patients. The following inclusion criteria were met: unilateral radicular pain in an upper extremity, age between 18 and 65 years, pain duration of at least 3 months, failure to respond to at least 6 weeks of conservative management (including medical and physical therapy), MRI findings consistent with a herniated disc correlating with patient’s signs and symptoms, minimum pain intensity score of 4 on the NRS. Patients with any of the following were excluded from the study: refusal to provide an informed consent, clinical or imaging evidence of cervical cord compression, psychological disorders, unstable medical conditions, prior cervical spine surgery, presence of contraindications for epidural access (coagulopathy, infection, allergy to study medications, patient refusal), CESI within the past 6 months, and pregnancy.

*Randomization and blinding*

 Twenty-six patients with unilateral radicular upper extremity pain were selected. Using a random allocation software, patients were randomly allocated into one of the two groups; MIL-CESI (n=13) and PSIL-CESI (n=13). Random numbers were kept in sealed envelopes. Both the patients and the physicians in charge of recording the data were blind to the groups to which the patients were allocated. The envelopes were opened by the physician performing the procedure at the time of procedure. Inevitably the proceduralists were aware of the routes they would choose to access the epidural space according to the number they drew out of the envelops. Proceduralists had no role in data collection, recording and analysis.

*CESI Procedures*

**Fig. 1:**
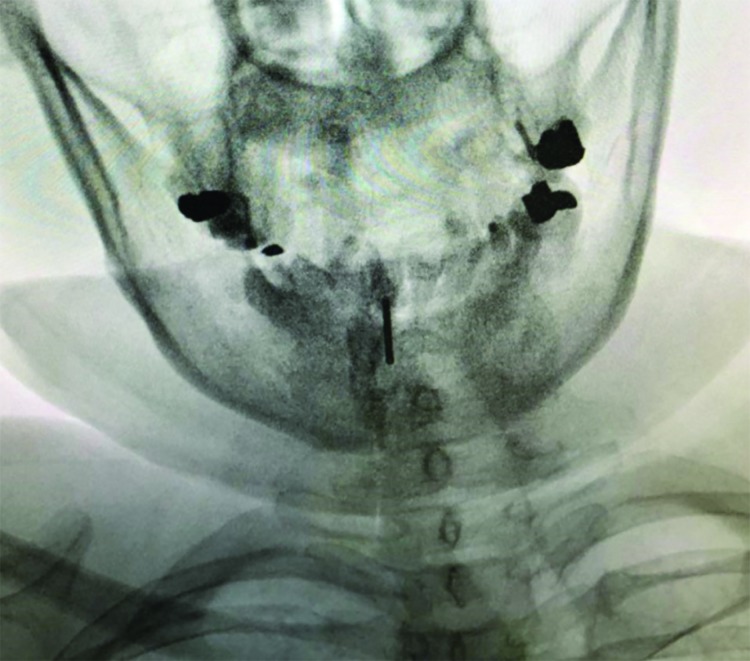
Parasagittal needle trajectory at C5-C6 interspace

**Fig. 2:**
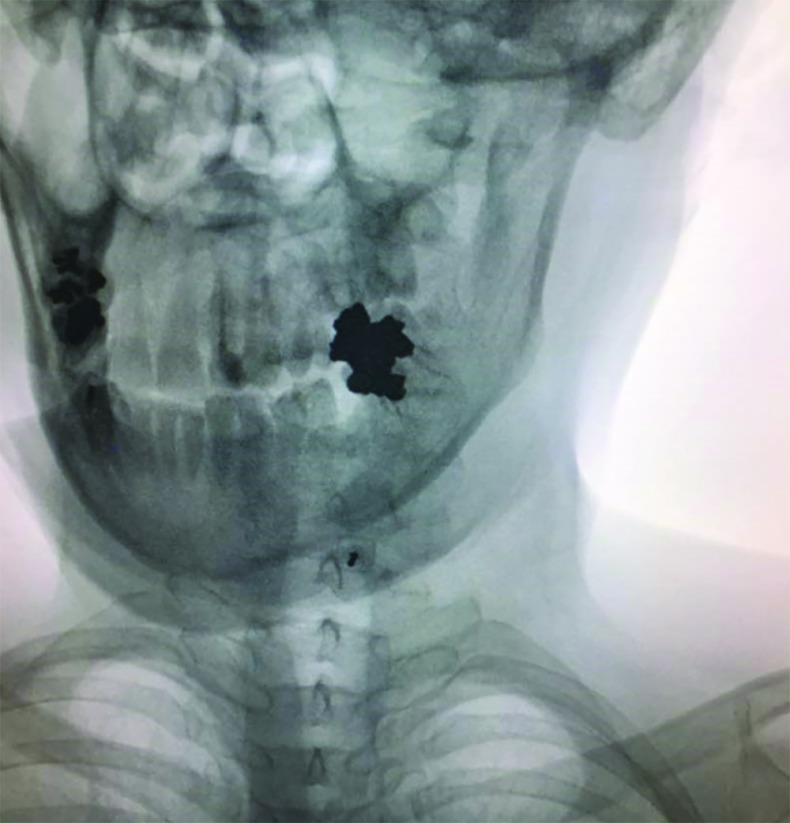
Parasagittal needle trajectory at C7-T1 interspace

**Fig. 3:**
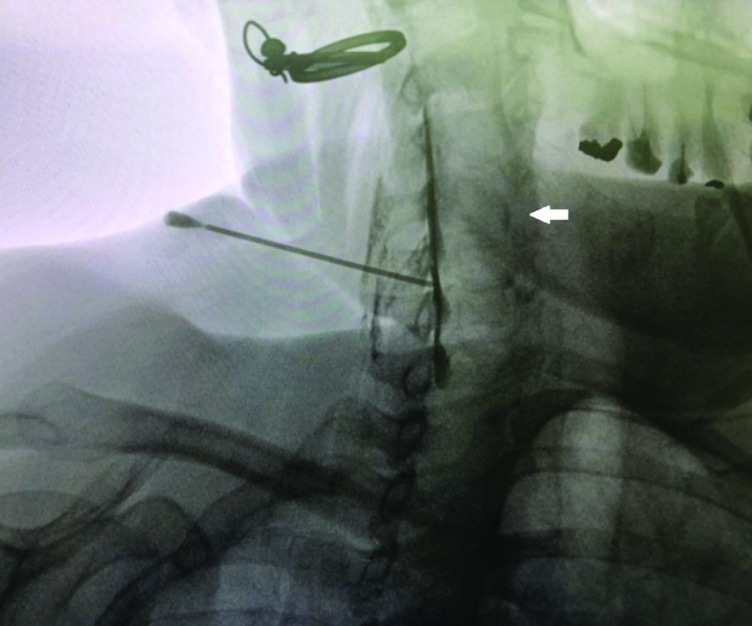
Confirmatory contralateral oblique (45○) view of radiocontrast distribution. Solid white arrow pointing to an ipsilateral fine crescent of radiocontrast

**Fig. 4:**
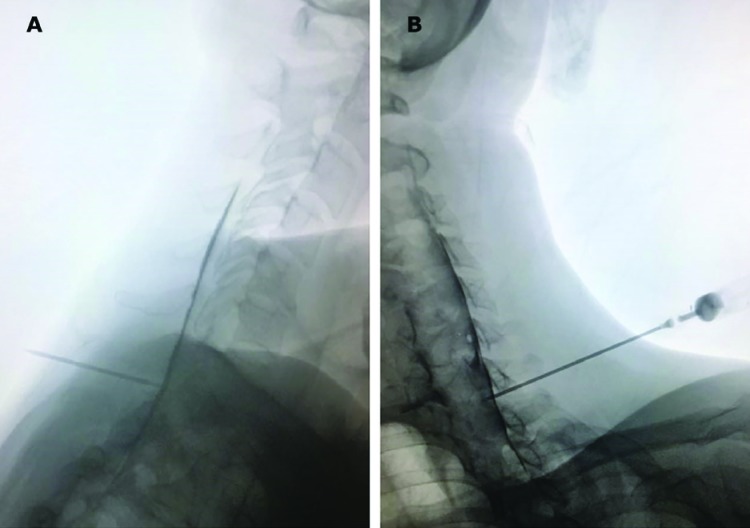
Lateral (A) and contralateral oblique (B) views of contrast spread of the same patient as in figure 3 (needle advanced through C7-T1 interspace)

 Procedures were performed in two academic medical centers. Proceduralists were either faculties certified in pain management subspecialty or senior in-training fellows of pain management subspecialty. Upon arriving to the pain OR, intravenous access was established for each patient. Patients assumed prone position on a fluoroscopy table. Monitoring included noninvasive blood pressure measurement, pulse oximetry and electrocardiography. The posterior cervical region was prepared with povidone-iodine 10% and draped in a sterile manner. Using fluoroscopic imaging needle entry points either at the C7-T1, C6-C7 or C5-C6 levels were selected. C5-C6 interspace was avoided and only chosen if visualization of other more inferior interspaces was unsatisfactory. Lidocaine 1%, 3-4 mL was used for local anesthesia. Using a saline loss-of-resistance technique a 17-gauge 3.5 inch Tuohy needle was advanced to the epidural space. Anteroposterior fluoroscopic images were used to guide the needle in a midline or parasagittal trajectory in a coaxial manner. A parasagittal trajectory was defined as a needle course passing between the lateral edge of the cervical spinous process and the medial border of the lamina in an anteroposterior (AP) fluoroscopic view (Figures 1 and 2). A midline trajectory was defined as a course confined to the borders of the cervical spinous process in an AP fluoroscopic view. We used lateral fluoroscopic control views in the midline group and 45○ contralateral oblique control views in the parasagittal group in order to add to the safety of the procedure. Upon acquiring a loss-of-resistance and after negative aspiration for cerebrospinal fluid or blood, 2 mL of the radiocontrast agent (Ominpaque TM, GE Healthcare, UK) was injected and fluoroscopic images (AP, lateral and 45○ contralateral oblique) were taken to confirm the epidural distribution of the radiocontrast (Figures 3 and 4). Next, a 5 mL volume of dexamethasone 8 mg in bupivacaine 0.125% were incrementally injected. Patients were observed for 30 minutes before discharge from the clinic. 

**Fig. 5:**
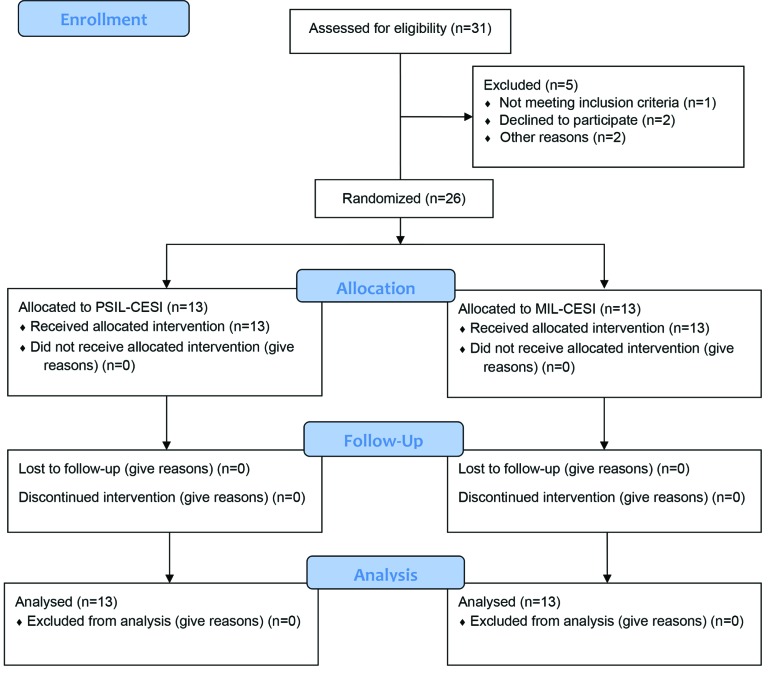
CONSORT flow diagram of the study

*Data Collection*

 The primary measured outcomes were changes in neck disability as measured by the NDI, and changes in pain intensity as measured by the NRS. Physicians who recorded the data had no role in performing the procedures. Data were gathered before the procedure (baseline) and one month after the procedure. The secondary measured outcome was the pattern of radiocontrast spread which was judged by the two physicians one of which, was the performing physician.

*Statistical analysis*

  Referring to the study conducted by Hashemi *et al*., [11] the following formula was used for calculation of the sample size:

The calculated sample size was 11 for each group. Considering attrition, we reached a sample size of 13 patients per group. Data analysis was performed using SPSS 20.0 software (Chicago, Illinois, USA). Chi-Square and Independent-Sample-T tests were used, and measurement outcomes were compared through the Repeated-Measurement ANOVA.  *P*-value less than 0.05 was considered to be statistically significant. 

## Results

All the patients finished the study and the flow diagram is summarized in Figure 5. Of the 26 enrolled patients, 15 (57.7%) were males and 11 (42.3%) were females. The mean age of the patients was 54.81 ± 6.72 (the age range was 46-66 years). The means for weight and height were 82.08 ± 8.12 kg (weight range of 70-95 kg) and 169.5 ± 8.86 cm (with a height range of 156-188 cm) respectively. Fifteen patients (57.7%) complained of right-sided radicular upper extremity pain and 11 patients (42.3%) complained of a left-sided radicular pain (Table 1).

 Regarding qualitative variables, there were no difference between the two groups (*p*=0.082) (Table 2).

**Table 1:** Distribution of the qualitative variables

**Variable**		**Frequency**	**Percent**
**Gender**	Male	15	57.7
	Female	11	42.3
**BMI category**	18.5-24.9	5	19.2
	25-29.9	12	46.2
	30-34.9	6	23.1
	35-39.9	3	11.5
**Painful side**	Right	15	57.7
	Left	11	42.3
**Radiocontrast agent**	Center	13	50
	Right	7	26.9
	Left	6	23.1
**Pain duration**	4 months	9	34.6
	5 months	8	30.8
	6 months	5	19.2
	7 months	4	15.4
**Herniated disc level**	C4-C5	1	3.8
	C5-C6	17	65.4
	C6-C7	8	30.8

**Table 2:** Chi-square test for categorized variables in MIL-CESI and PSIL-CESI groups

**Variable**		**Midline group**		**Parasagittal group**		**X2, ** **p-value**
		**Percent**	**Frequency**	**Percent**	**Frequency**	
**Gender**	Male	69.2	9	46.2	6	X2=1.418
	Female	30.8	4	53.8	7	P=0.214
**BMI category**	18.5-24.9	30.8	4	7.7	1	
	25-29.9	38.5	5	53.8	7	X2=2.467
	30-34.9	23.1	3	23.1	3	P=0.418
	35-39.9	7.7	1	15.4	2	
**Pain duration**	4 months	38.5	5	30.8	4	
	5 months	30.8	2	30.8	4	X2=0.311
	6 months	15.4	2	23.1	3	P=0.958
	7 months	15.4	2	15.4	2	
**Herniated disc level**	C4-C5	0	0	7.7	1	
	C5-C6	69.2	9	61.5	8	P=0.387
	C6-C7	30.8	4	30.8	4	
**Painful side**	Right	61.5	8	53.8	7	X2=0.158
	Left	38.5	5	46.2	6	P=0.5
**Distribution of radiocontrast**	Center	92.3	12	7.7	1	X2=18.97
	Right	0	0	53.8	7	P=0.001
	Left	7.7	1	38.5	5	

*Functional Improvement*

 Improvement in the NDI was seen in both groups one month after the procedures. Meanwhile, this improvement was significantly more pronounced in the PSIL-CESI group as compared to the MIL-CESI group (Table 3).

**Table 3:** Paired T-test regarding NDI PSIL-CESI and MIL-CESI groups before and after intervention

**Variable**	**Group**	**Mean**	**Std. Deviation**	**t**	**P value**
**NDI (PSIL-CESI group)**	Before intervention	25	5.598	4.311	0.001
	After intervention	16	7.257		
**NDI (MIL-CESI group)**	Before intervention	24	4.262	1.851	0.011
	After intervention	19.38	6.376		

*Effective Pain Relief*

 No significant difference in pain intensity was observed between the two groups before the interventions. Both groups experiences significant alleviation of pain. Reduction of pain intensity was more pronounced in patients in the PSIL-CESI group in comparison to patients in the MIL-CESI group (Table 4).

**Table 4:** Paired T-test regarding NSR in MIL-CESI and PSIL-CESI before and after intervention

**Variable**	**Group**	**Mean**	**Std. Deviation**	**t**	**P value**
**Parasagittal NSR**	Before intervention	5.92	0.760	8.379	0.001
	After intervention	2.92	1.441		
**Midline NSR**	Before intervention	6.08	0.954	5.007	0.001
	After intervention	3.00	1.958		

*Patterns of radio-contrast Spread*

 In all 13 patients of the MIL-CESI group, the pattern of radiocontrast spread was predominantly midline. Among patients in the PSIL-CESI group, in 7 cases (53.8%) radiocontrast spread was predominantly ipsilateral right-sided and in 6 cases (46.1%) predominantly ipsilateral left-sided. With the PSIL-CESI approach in 38% (5/13) of instances a ventral spread of radiocontrast was observed as opposed to 0.7% (1/13) with the MIL-CESI approach (Table 5).

**Table 5:** Patterns of radio-contrast spread in PSIL-CESI and MIL-CESI groups

	**Intervention Groups**	
**Radio-contrast Spread Pattern**	**PSIL-CESI group**	**MIL-CESI group**
**Predominantly ipsilateral left**	6 (46.1%)	0 (0%)
**Predominantly midline**	0 (0%)	13 (100%)
**Predominantly ipsilateral right**	7 (53.8%)	0 (0%)
**Ventral **	5 (38%)	1 (0.7%)

## Discussion

Cervical epidural steroid injections are among the most prevalent interventions used in the management of cervical radicular pain due to disc herniation. This procedure is especially valuable in those who are poor candidates for surgery [1-5]. We compared the clinical efficacy of two different approaches to CESI in a parallel randomized double-blind clinical trial; MIL-CESI and PSIL-CESI. Both groups showed clinically significant improvement regarding to both pain intensity and degree of functional disability of cervical origin. The PSIL-CESI group, however, showed superior outcome after 1 month of the procedure.

 Targeted approach to the delivery of medications into the epidural space has been investigated in the lumbar region. The rationale behind taking a TF approach to access the epidural space has been the hypothesis that ventral spread of the injectate addresses the pathology site more thoroughly [11, 14-16]. Reports of devastating complications with the TF approach have urged practitioners to develop new techniques avoiding the neural foramina. Complications such as arterial spasm, arterial dissection, nerve root trauma, spinal cord trauma, brainstem infarction, and death have been attributed to this route of epidural access [17, 18]. PSIL approach has been advocated as an alternative to the MIL approach in the lumbar region. Higher ventral spread of radiocontrast and superior efficacy of the PSIL approach in comparison to the MIL approach in unilateral lower extremity radicular pain has been shown in a number of studies [11, 12]. KD Candido *et al*., [13] observed a striking 100% percent ventral spread of radiocontrast [5 mL] in the lateral PSIL-ESI group in the lumbar region as opposed to a 75% ventral spread in the MIL-ESI group.

 Recently two groups of investigators have introduced alternative techniques; E. Choi *et al*., [1] introduced the modified paramedian technique for targeted delivery of the injectate into the cervical epidural space and compared it with the TF route of epidural delivery. Despite the fact that ventral spread of radiocontrast was significantly higher in the modified paramedian group, no clinically significant difference in the efficacy of the two approaches was observed at any point during the study time span. Zachary L McCormick *et al*., [19] compared the standard interlaminar CESI with targeted CESI by leading an epidural catheter to the site corresponding to the radicular pathology after a midline interlaminar needle insertion. Although they observed meaningful clinical improvement in both groups, they did not report any outcome difference between the group subjects. 

Although we observed clinically significant improvements in the scores of the NDI and the NRS in both groups, those improvements were significantly more pronounced in the PSIL-CESI group. Radiocontrast distribution (2 mL) to the ventral epidural space was seen in 38% (5/13) of patients in the PSIL-CESI group and only in 0.7% (1/13) in the MIL-CESI group. Reports of radiocontrast spread to the ventral epidural space in the cervical region are highly variable; Jatindar Gill *et al*., [20] performed a three-dimensional analysis of cervical contrast spread pattern. They did not report any instance of ventral spread of radiocontrast in their study. Accordingly, they warned that with low volumes of radiocontrast (2 mL) visualization of radiocontrast in the ventral epidural space should raise concerns regarding a subarachnoid spread. In contrary to those observations E Choi *et al*., [1] reported a 90.4% anterior contrast spread using 2 mL of radiocontrast through a modified paramedian interlaminar approach to the cervical epidural space. Difference in types of cervical pathologies in study subjects may explain such variabilities.

Due to the incidence of devastating complications attributed to the TF approach, this route of access to the cervical epidural space cannot be advocated. Considering the fact that the depth of the cervical epidural space at C7 is as little as 1.5-2 mm, the previously mentioned modified paramedian interlaminar approach seems technically demanding and potentially unsafe [1,21]. We propose the PSIL-CESI [ipsilateral to the radicular symptoms] as an effective and safe alternative to the TF, modified paramedian and MIL approaches.

## Conflict of interest:

The authors declared that there was no conflict of interest in this study.
